# Supplementary material for: A novel deep neural network structure for software fault prediction
Source: PeerJ Comput Sci. 2024 Oct 17;10:e2270. doi: 10.7717/peerj-cs.2270 (PMC11623021; doi:10.7717/peerj-cs.2270)
Supplement: Supplemental Information 1 [file peerj-cs-10-2270-s001.docx]

**Table S1:** Software metrics of the Bughunter dataset [19]

| **Abbreviation** | **Full name** |
| --- | --- |
| CLOC | Comment Lines of Code |
| LOC | Lines of Code |
| LLOC | Logical Lines of Code |
| NL | Nesting Level |
| NLE | Nesting Level Else-If |
| NII | Number of Incoming Invocations |
| NOI | Number of Outgoing Invocations |
| CD | Comment Density |
| TCD | Total Comment Density |
| TCLOC | Total Comment Lines of Code |
| NOS | Number of Statements |
| TLOC | Total Lines of Code |
| CC | Clone Coverage |
| CCL | Clone Classes |
| CCO | Clone Complexity |
| CI | Clone Instances |
| CLC | Clone Line Coverage |
| CLLC | Clone Lines of Code |
| LDC | Lines of Duplicated Code |
| LLDC | Logical Lines of Duplicated Code |
