# Supplementary figures and images for: A novel deep neural network structure for software fault prediction
Source: PeerJ Comput Sci. 2024 Oct 17;10:e2270. doi: 10.7717/peerj-cs.2270 (PMC11623021; doi:10.7717/peerj-cs.2270)

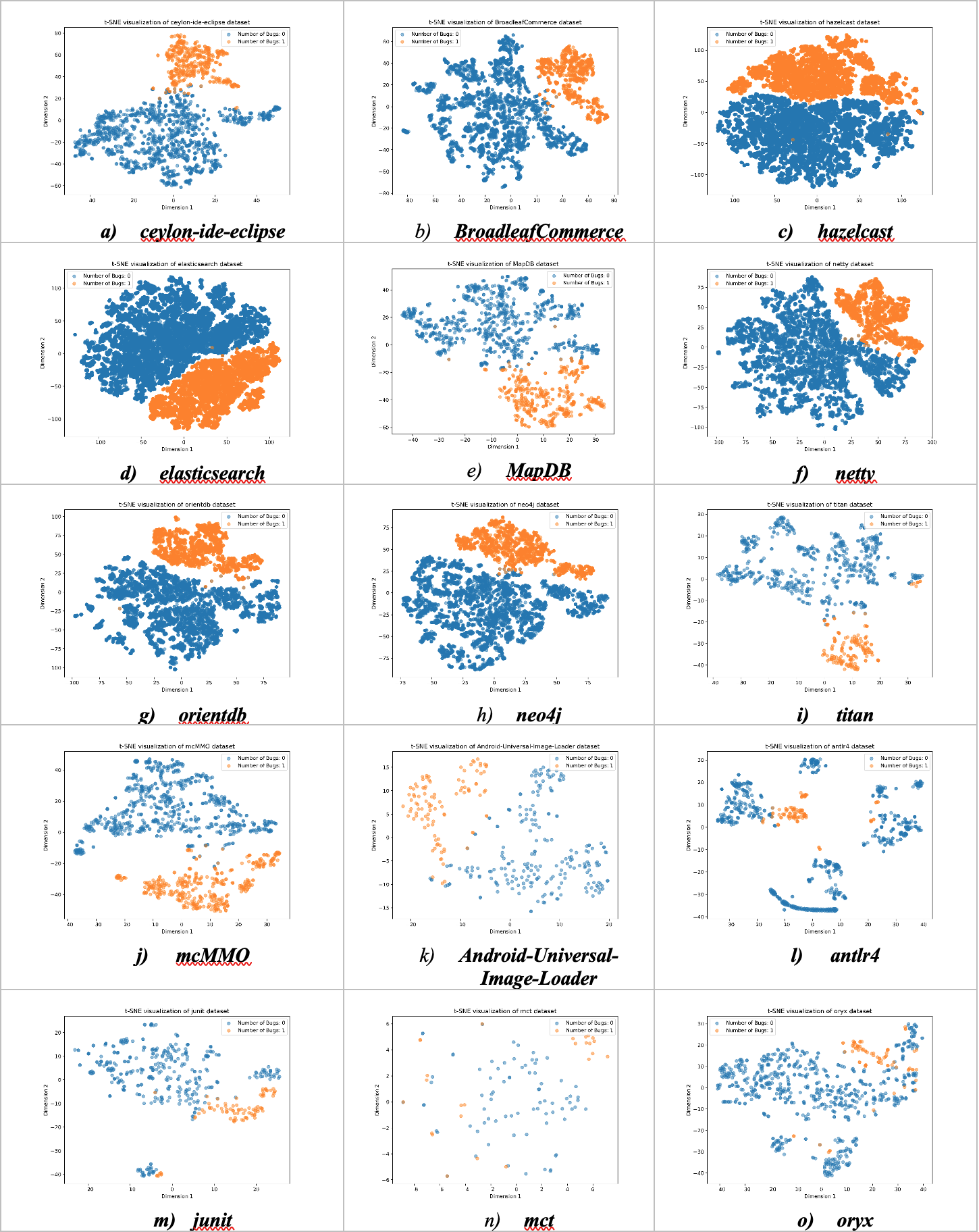

Supplement: Supplemental Information 2 — We utilized the t-distributed Stochastic Neighbor Embedding (t-SNE) technique to plot the data points of each class.The x-axis and y-axis of each plot represent the two dimensions obtained from reducing the high-dimensional data to two dimensions using t-SNE. Each point in the scatter plot corresponds to an instance of the dataset, and its color is associated with the label of that instance, which is either faulty or non-faulty. The BugHunter dataset comprises source code metrics and clone metrics. From the comprehensive list of software metrics presented at the method level, Table S1 displays a subset of them for simplicity. [file peerj-cs-10-2270-s002.png]
